# Supplementary material for: Pachychoroid Spectrum Diseases in Patients with Cushing’s Syndrome: A Systematic Review with Meta-Analyses
Source: J Clin Med. 2022 Jul 29;11(15):4437. doi: 10.3390/jcm11154437 (PMC9369356; doi:10.3390/jcm11154437)
Supplement: Supplementary file 1 [file jcm-11-04437-s001.zip › Supplementary File S1.pdf]

Supplementary File S1. Details of literature search.

PubMed:

History and Search Details

Download
Delete

Search	Actions	Details	Query	Results	Time
#1			Search: **("Cushing" OR "Cushings" OR "Cushing's" OR "Cushing Syndrome"[Mesh]) AND "Optical Coherence Tomography"** Sort by: **Most Recent** ("Cushing"[All Fields] OR "Cushings"[All Fields] OR "Cushing's"[All Fields] OR "Cushing Syndrome"[MeSH Terms]) AND "Optical Coherence Tomography"[All Fields]	19	04:56:40

EMBASE:

Search History
(7searches found)

Contract	# ▲	Searches	Results	Type	Actions	Annotations
	1	Cushing.mp. [mp=title, abstract, heading word, drug trade name, original title, device manufacturer, drug manufacturer, device trade name, keyword heading word, floating subheading word, candidate term word]	22792	Advanced	Display Results More	
	2	Cushings.mp. [mp=title, abstract, heading word, drug trade name, original title, device manufacturer, drug manufacturer, device trade name, keyword heading word, floating subheading word, candidate term word]	14193	Advanced	Display Results More	
	3	Cushing's.mp. [mp=title, abstract, heading word, drug trade name, original title, device manufacturer, drug manufacturer, device trade name, keyword heading word, floating subheading word, candidate term word]	14189	Advanced	Display Results More	
	4	*Cushing syndrome/ or *Cushing disease/	11319	Advanced	Display Results	

				More		
5	"Optical coherence tomography".mp. [mp=title, abstract, heading word, drug trade name, original title, device manufacturer, drug manufacturer, device trade name, keyword heading word, floating subheading word, candidate term word]	85766	Advanced	More	Display Results	
6	1 or 2 or 3 or 4	25022	Advanced	More	Display Results	
7	5 and 6	46	Advanced	More	Display Results	

Combine with:

Web of Science Core Collection, BIOSIS Previews, Current Contents Connect, Data Citation Index, Derwent Innovations Index, KCI-Korean Journal Database, SciELO Citation Index, Zoological Record:

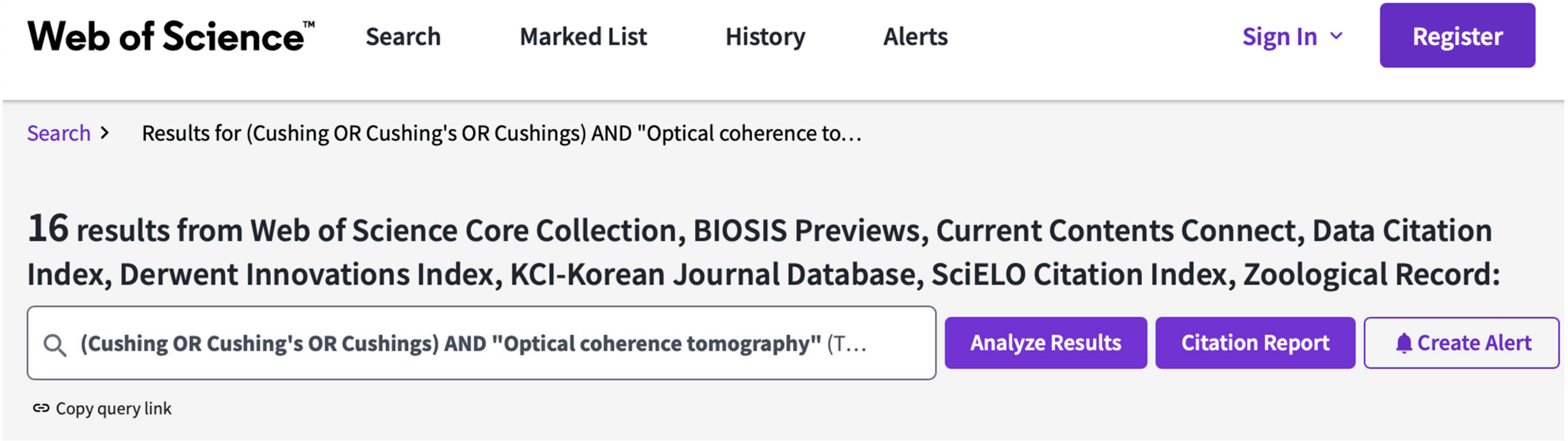

The Cochrane Library:

Advanced Search

Search | **Search manager** | Medical terms (MeSH) | PICO search

Save this search | View/Share saved searches | Search help

Print search history

#1	("Cushing disease") OR ("Cushing syndrome") OR ("Cushing's syndrome") OR ("Cushings syndrome") OR (Cushing)<br>(Word variations have been searched)	Limits	729
#2	"Optical coherence tomography"	Limits	3989
#3	#1 AND #2	Limits	1
#4	Type a search term or use the S or MeSH buttons to compose	Limits	N/A

Clear all

Highlight orphan lines
