# Supplementary material for: Pachychoroid Spectrum Diseases in Patients with Cushing’s Syndrome: A Systematic Review with Meta-Analyses
Source: J Clin Med. 2022 Jul 29;11(15):4437. doi: 10.3390/jcm11154437 (PMC9369356; doi:10.3390/jcm11154437)
Supplement: Supplementary file 1 [file jcm-11-04437-s001.zip › Supplementary Table S1.pdf]

Supplementary Table S1. Sensitivity analysis of the weighted mean difference summary estimate in subfoveal choroidal thickness between patients with Cushing's syndrome and matched healthy controls.

Excluded study	Pooled WMD	LCI 95%	HCI 95%	Cochran Q	p	I^2
Eymard et al. 2021	62,81	24,73	100,88	11,37	0,009882	73,62
Karaca et al. 2017	52,31	0,27	104,35	21,25	9,34E-05	85,88
Lassandro et al. 2022	51,66	3,92	99,40	21,52	8,21E-05	86,06
Wang et al. 2019	48,94	-1,86	99,75	21,60	7,9E-05	86,11
Abalem et al. 2016	31,16	7,31	55,01	4,68	0,196651	35,92
