# Supplementary material for: Pachychoroid Spectrum Diseases in Patients with Cushing’s Syndrome: A Systematic Review with Meta-Analyses
Source: J Clin Med. 2022 Jul 29;11(15):4437. doi: 10.3390/jcm11154437 (PMC9369356; doi:10.3390/jcm11154437)
Supplement: Supplementary file 1 [file jcm-11-04437-s001.zip › Supplementary Table S2.pdf]

Supplementary Table S2. Sensitivity analysis of the summary estimate prevalence of pachychoroid pigment epitheliopathy.

Excluded study	Pooled Prevalence	LCI 95%	HCI 95%	Cochran Q	p	I^2
Abalem et al. 2016	25,4%	10,9%	43,2%	4,15	0,041754	75,88
Eymard et al. 2021	20,2%	1,2%	49,6%	5,79	0,016119	82,73
Lassandro et al. 2022	15,6%	8,3%	24,6%	0,77	0,381506	0,00
