# Supplementary material for: Pachychoroid Spectrum Diseases in Patients with Cushing’s Syndrome: A Systematic Review with Meta-Analyses
Source: J Clin Med. 2022 Jul 29;11(15):4437. doi: 10.3390/jcm11154437 (PMC9369356; doi:10.3390/jcm11154437)
Supplement: Supplementary file 1 [file jcm-11-04437-s001.zip › Supplementary Table S3.pdf]

Supplementary Table S3. Sensitivity analysis of the summary estimate prevalence of central serous chorioretinopathy.

Excluded study	Pooled Prevalence	LCI 95%	HCI 95%	Cochran Q	p	I 2
Eymard et al. 2021	11,7%	4,4%	21,6%	7,17	0,066751	58,14
Karaca et al. 2017	8,4%	0,0%	24,8%	19,20	0,000249	84,37
Abalem et al. 2016	7,0%	0,0%	20,6%	19,16	0,000253	84,34
Wang et al. 2019	7,9%	0,0%	20,2%	16,85	0,00076	82,19
Brinks et al. 2021	4,8%	0,2%	13,3%	10,84	0,012639	72,32
