# Supplementary material for: Pachychoroid Spectrum Diseases in Patients with Cushing’s Syndrome: A Systematic Review with Meta-Analyses
Source: J Clin Med. 2022 Jul 29;11(15):4437. doi: 10.3390/jcm11154437 (PMC9369356; doi:10.3390/jcm11154437)
Supplement: Supplementary file 1 [file jcm-11-04437-s001.zip › Supplementary Table S4.pdf]

Supplementary Table S4. Sensitivity analysis of the summary estimate prevalence of polypoidal choroidal vasculopathy.

Excluded study	Pooled Prevalence	LCI 95%	HCI 95%	Cochran Q	p	I 2
Abalem et al. 2016	3,2%	0,8%	6,8%	0,75	0,688448	0,00
Brinks et al. 2021	3,2%	0,8%	6,8%	0,75	0,688448	0,00
Lassandro et al. 2022	2,2%	0,1%	6,2%	1,10	0,576052	0,00
Eymard et al. 2021	2,1%	0,1%	5,9%	0,89	0,642218	0,00
