# Supplementary figures and images for: Pachychoroid Spectrum Diseases in Patients with Cushing’s Syndrome: A Systematic Review with Meta-Analyses
Source: J Clin Med. 2022 Jul 29;11(15):4437. doi: 10.3390/jcm11154437 (PMC9369356; doi:10.3390/jcm11154437)

Standard error

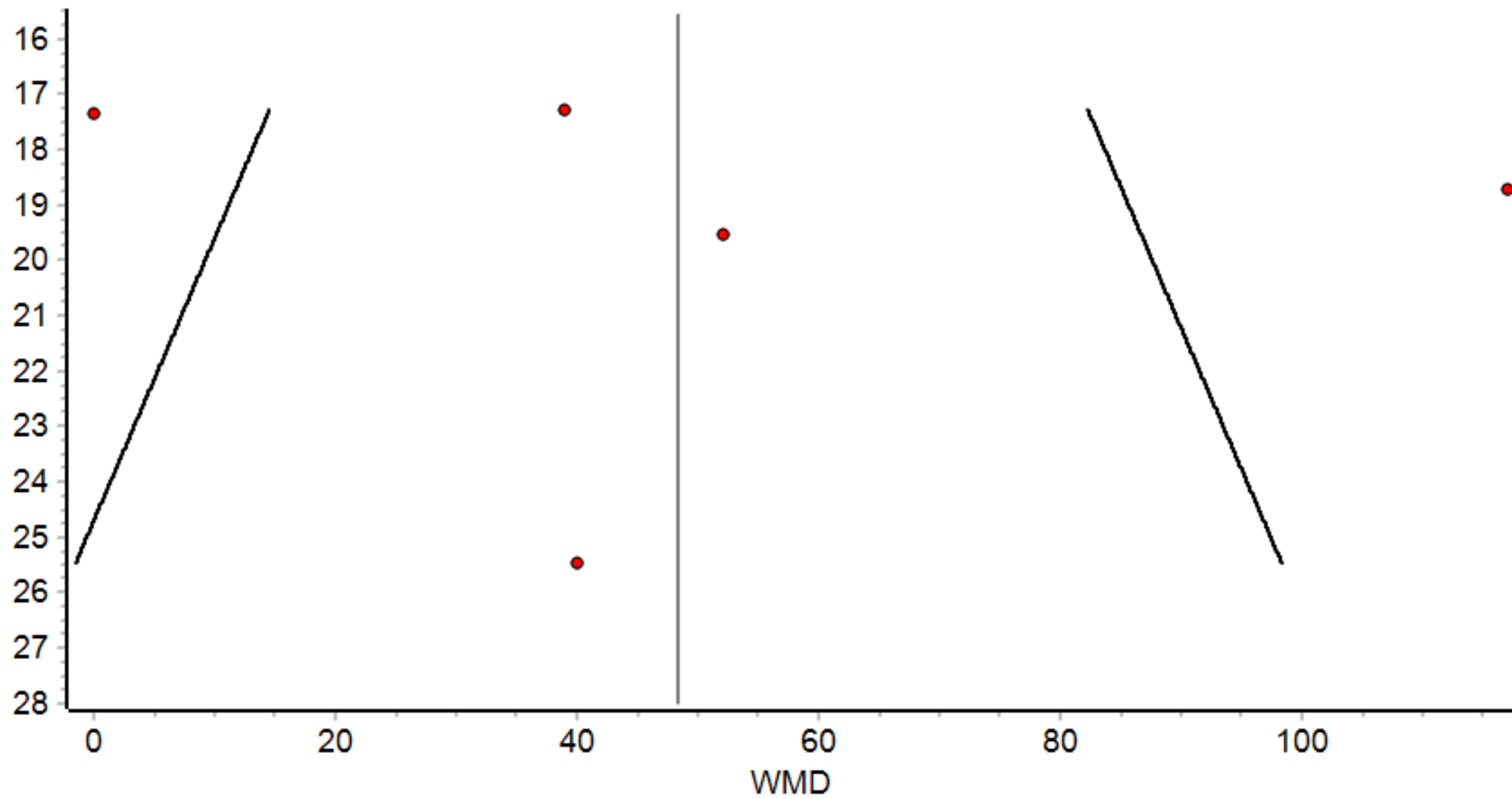

Supplement: Supplementary file 1 [file jcm-11-04437-s001.zip › Supplementary Figure S1.pdf]

Standard error

0,12  
0,13  
0,14  
0,15  
0,16  
0,17  
0,18  
0,19  
0,2  
0,21  
0,22  
0,23

0,7

0,8

0,9

1

1,1

1,2

1,3

1,4

Double Arcsin Prevalence

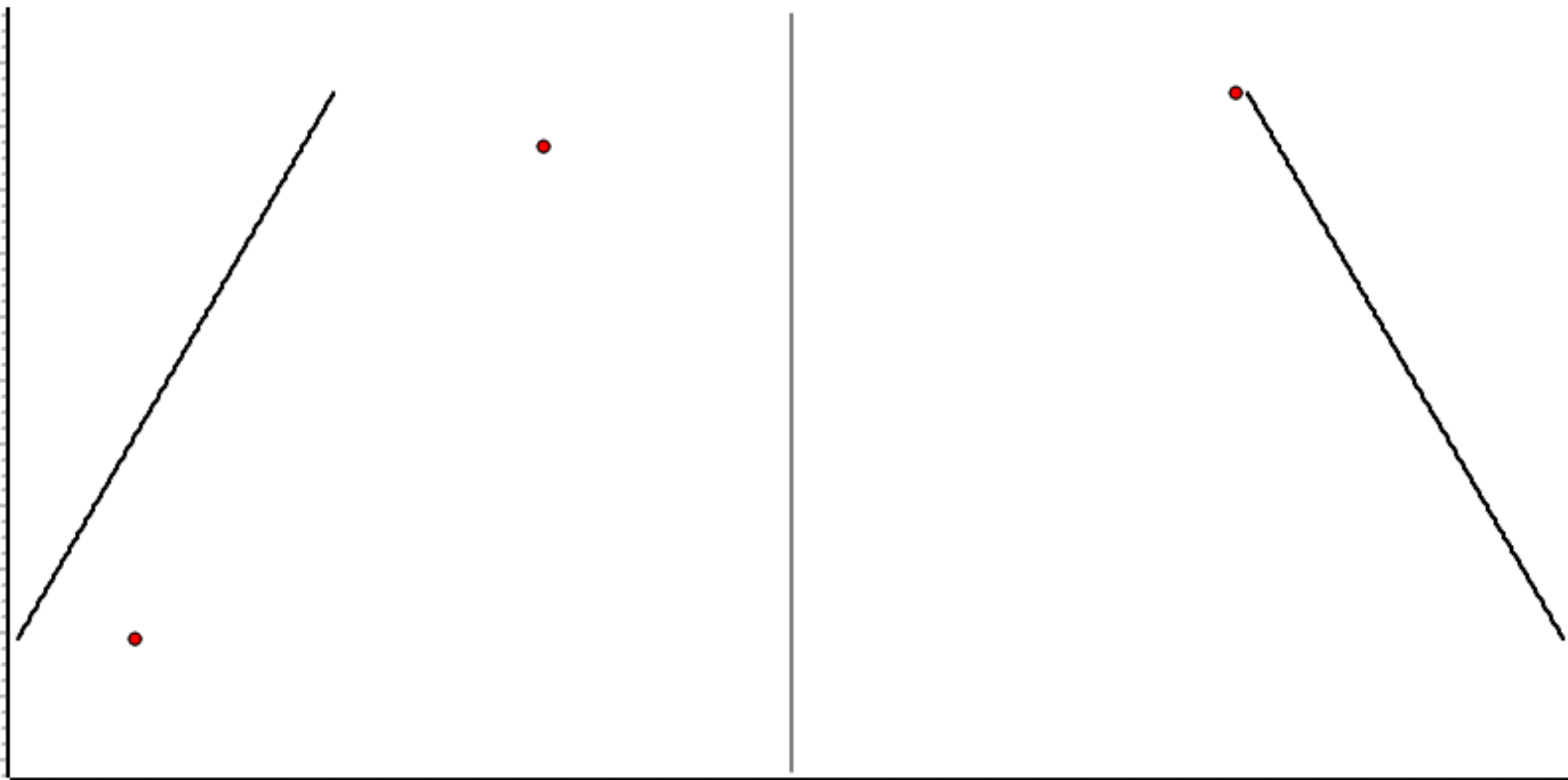

Supplement: Supplementary file 1 [file jcm-11-04437-s001.zip › Supplementary Figure S2.pdf]

Standard error

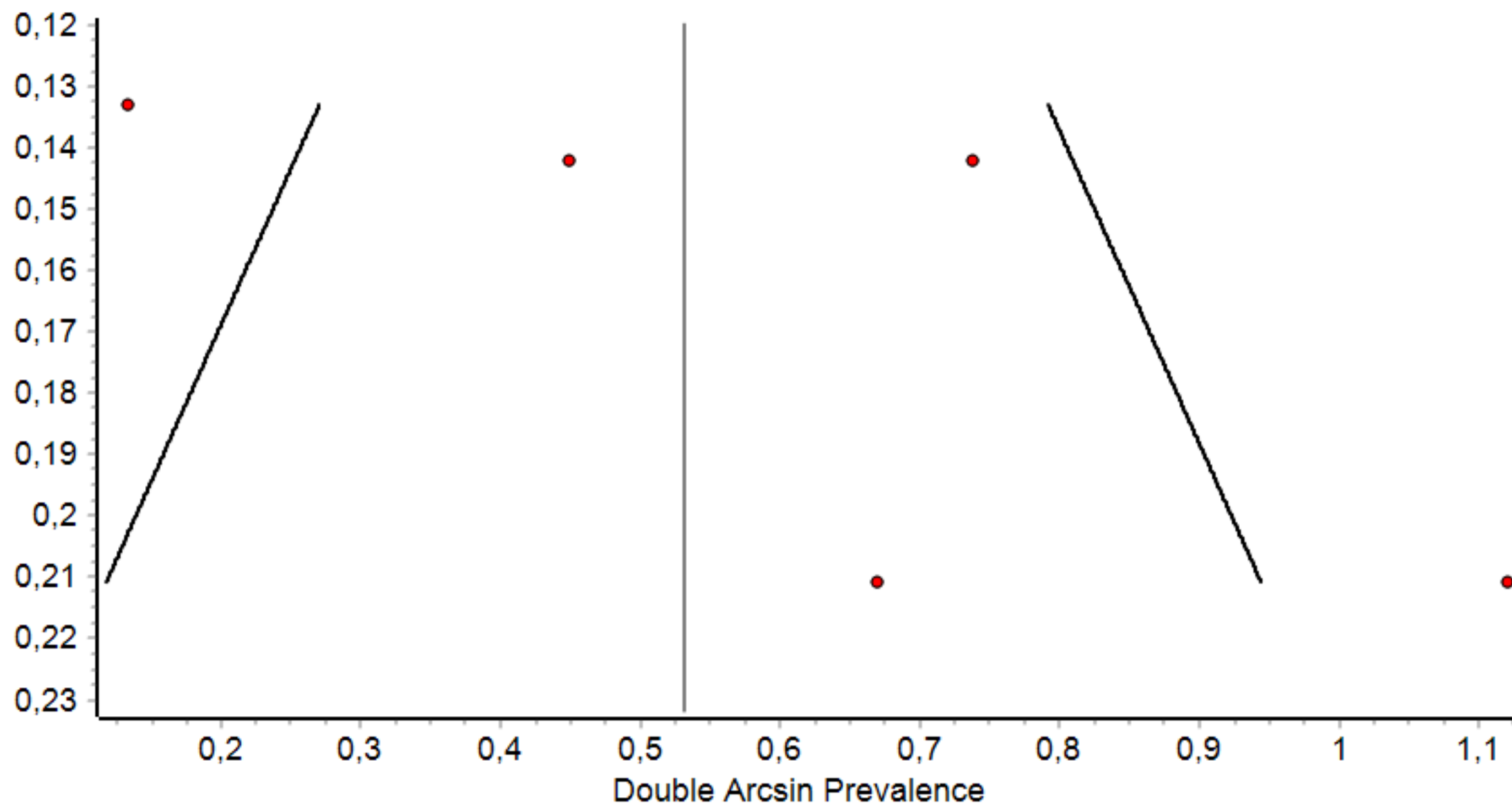

Supplement: Supplementary file 1 [file jcm-11-04437-s001.zip › Supplementary Figure S3.pdf]

Standard error

0,12  
0,13  
0,14  
0,15  
0,16  
0,17  
0,18  
0,19  
0,2  
0,21  
0,22  
0,23

0

0,1

0,2

0,3

0,4

0,5

0,6

0,7

Double Arcsin Prevalence

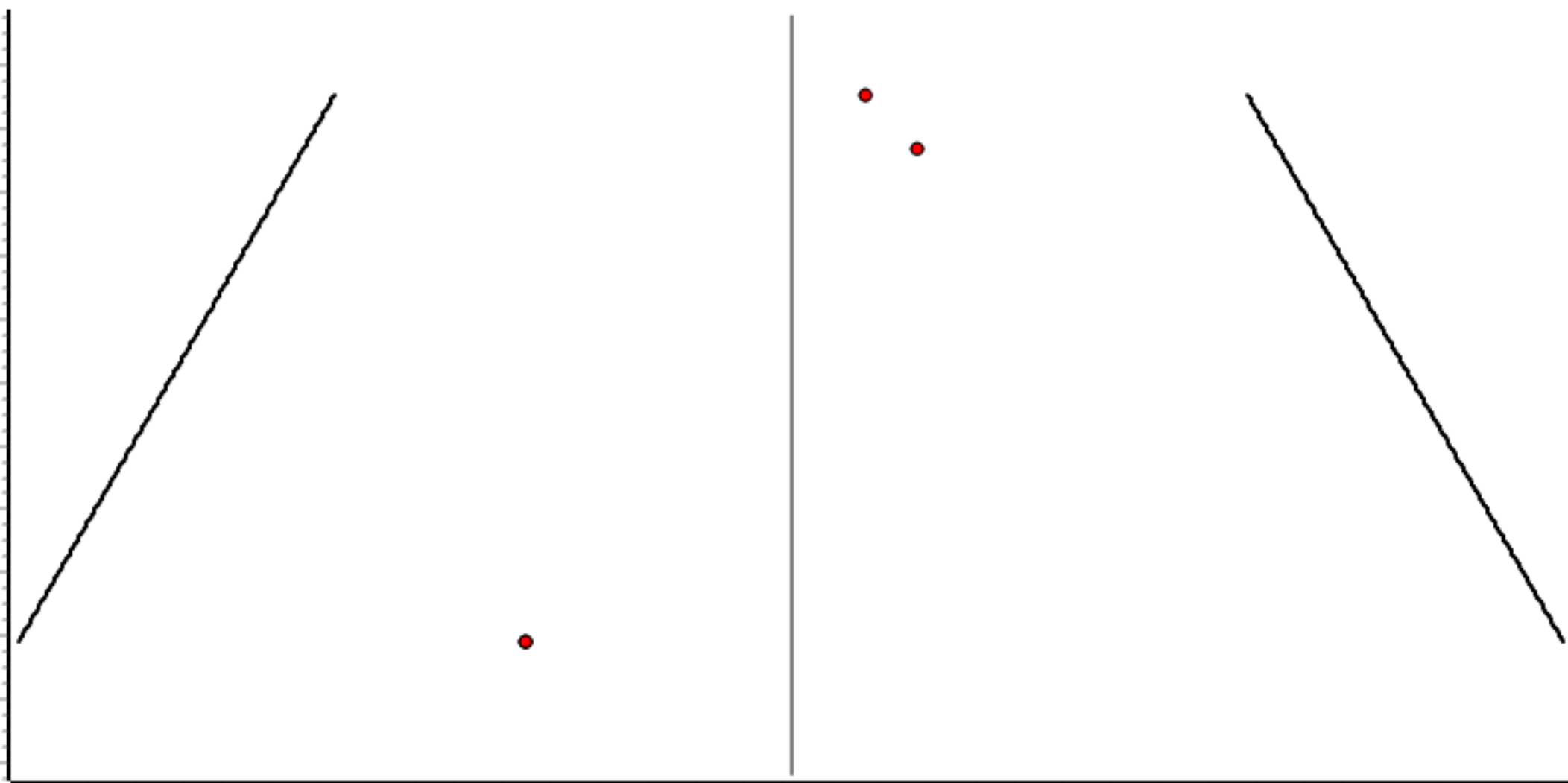

Supplement: Supplementary file 1 [file jcm-11-04437-s001.zip › Supplementary Figure S4.pdf]
